# Colchicine Therapy for Glenohumeral Osteoarthritis: A Case Report

**DOI:** 10.7759/cureus.59181

**Published:** 2024-04-27

**Authors:** Oman Sadik, Sophia Tahir, Anum Sahibzada, Chinenye Iguh, Virginia Ezenwa, Sravani Bhavanam

**Affiliations:** 1 Family Medicine, Jackson Park Hospital, Chicago, USA; 2 Internal Medicine, Windsor University School of Medicine, Cayon, KNA; 3 Internal Medicine, Saint James School of Medicine, The Quarter, AIA; 4 Medicine, Windsor University School of Medicine, Cayon, KNA; 5 Internal Medicine, Sri Devaraj Urs Medical College, Kolar, IND

**Keywords:** gout disease, medication for pain management, shoulder joint pain, colchicine treatment, glenohumeral osteoarthritis

## Abstract

Osteoarthritis management primarily focuses on targeting pain. Conventional modalities for pain management include acetaminophen, non-steroidal anti-inflammatory drugs (NSAIDs), and intra-articular corticosteroid injections. However, these approaches may provide minimal pain relief and can be contraindicated for some patients, highlighting the ongoing need for alternative pain management. Colchicine, commonly used in the management of gout, has emerged as a potential option for pain management in osteoarthritis. There are implications of colchicine use for knee and hand osteoarthritis but remains inconclusive. In this context, we present a case of a 68-year-old diabetic woman with glenohumeral osteoarthritis and associated right shoulder pain. Due to minimal pain relief from previous treatments, the patient was given a combination trial of colchicine and acetaminophen for three months. After completion of this treatment, the patient experienced significant pain relief and improved functionality. The aim of this case is to highlight the efficacy of colchicine as a possible treatment option for managing shoulder pain in osteoarthritis.

## Introduction

Osteoarthritis (OA) is ranked as the 11th most debilitating disease worldwide, affecting 80% of the elderly population in the United States alone [1]. OA is a chronic degenerative joint condition characterized by a neutrophil-mediated deterioration of articular cartilage, synovial inflammation, and subchondral bone alterations [2]. Neutrophils infiltrate the synovial tissue, releasing pro-inflammatory cytokines such as interleukin-6 (IL-6), tumor necrosis factor-alpha (TNF-alpha), and protease enzymes like neutrophil elastase, causing significant damage to the joints [3]. This primarily affects weight-bearing joints like the knees and hips, but also extends to the shoulders, hands, and feet [4]. In fact, glenohumeral OA is the most common cause of shoulder pain in the elderly population [5].

Targeting inflammation is crucial in OA treatment to reduce the severity of the disease. Current therapeutic approaches primarily focus on pain management and lifestyle modifications, where initial management involves oral non-steroidal anti-inflammatory drugs (NSAIDs) and acetaminophen [6]. Other approaches include topical NSAIDs, physical therapy, and intra-articular corticosteroid (IACS) injections [6]. However, many patients are left with suboptimal outcomes due to other comorbidities, adverse effects, and limitations of currently effective therapies, promoting a growing interest in exploring alternative treatments for pain.

Colchicine, obtained from the autumn crocus plant, has successfully managed other joint-affecting conditions like gout and familial Mediterranean fever due to its significant anti-inflammatory properties [7]. Its potential to inhibit microtubule polymerization and neutrophil chemotaxis has led to investigations into its role as a treatment modality for OA [8]. While the efficacy of colchicine in the context of OA treatment remains limited, studies have reported its potential benefits for management of OA of the hands and knees when given in combination with other agents [9]. Given the heterogeneity of these findings, we present a unique case of a 68-year-old woman given colchicine for pain management for her right glenohumeral OA, which has not been previously reported in the literature. 

## Case presentation

A 68-year-old woman presented to the clinic with complaints of right shoulder pain for one month. Her medical history included a five-year history of OA that initially affected her right knee, progressed to her hip, and finally manifested as pain in her right shoulder. Additionally, the patient had a medical history of type II diabetes, controlled with dietary modifications and diabetic medication. Her previous OA management consisted of a combination of acetaminophen 1000 mg and ibuprofen 600 mg daily for pain as needed, along with diclofenac sodium 1% topical gel for her affected joints, but this regimen provided her temporary relief. Weekly physical therapy sessions were also recommended, while they were effective for a varied duration, limitations in transportation prevented the patient from adhering to this management plan. As her shoulder pain continued to worsen, she was given IACS injections in the right subacromial space at six-month intervals during 2022 and 2023. While the pain relief was effective, her uncomfortable experiences of high glucose levels as a side effect and the physical pain of the injection, outweighed the benefits and she requested to discontinue. 

Despite continuous use of her oral and topical management, she currently rates her pain intensity as 7 out of 10 on the visual analog scale (VAS). Physical examination revealed a restricted range of motion with extension and abduction above 90 degrees. Palpation revealed tenderness surrounding the glenohumeral joint, along with minimal tenderness over the acromioclavicular joint and biceps tendon, consistent with osteoarthritis pain patterns. Manual muscle testing maneuvers revealed mild weakness of the infraspinatus and supraspinatus muscles. The patient emphasizes limitations in daily activities such as reaching over her head and lifting heavy objects. A comprehensive metabolic panel (CMP) and magnetic resonance imaging (MRI) imaging were ordered.

**Figure 1:**
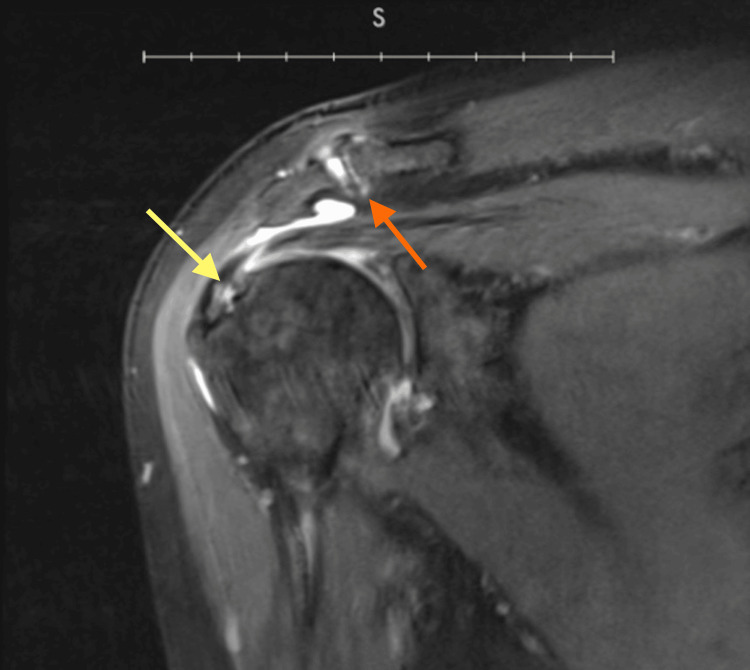
Coronal T1-weighted MRI without contrast Thickening of the supraspinatus tendon and intrasubstance signal consistent with tendinopathy is shown, along with partial-thickness articular surface tear of the supraspinatus and delamination of tendon fibers (yellow arrow). Subacromial bone spur and hypertrophic changes are seen (orange arrow).

**Figure 2:**
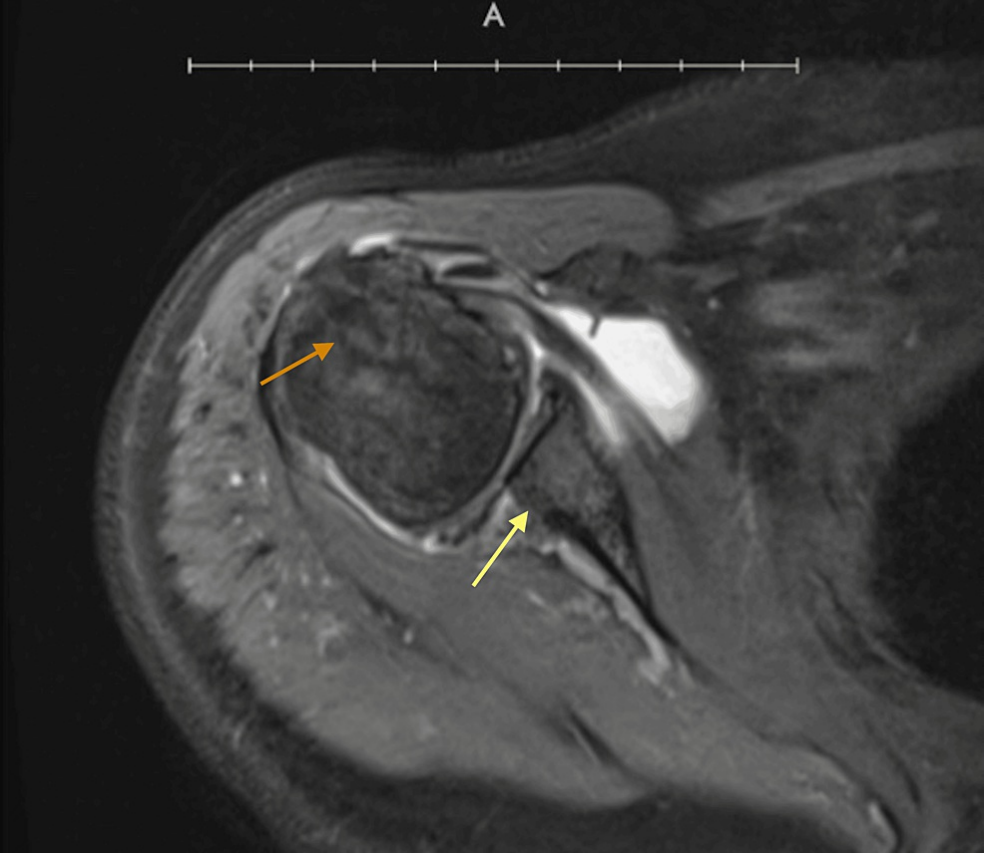
Axial MRI without contrast Chondral thinning of the humeral head (orange arrow). Full-thickness cartilage loss at the posterior inferior glenoid and subchondral cystic change is also noted (yellow arrow).

The erythrocyte sedimentation rate (ESR) was 24 mm/hr and C-reactive protein (CRP) was 13 mg/L. MRI imaging revealed marked tendinosis, interstitial tears of the intracapsular biceps tendon, tendinopathy with partial-thickness insertional tears and delamination of multiple tendons (Figure 1); hypertrophic degeneration of acromioclavicular joint and subacromial bone spur (Figure 1); Degenerative arthritis with areas of full thickness chondral loss at the posterior inferior glenoid was evident, along with the humeral head (Figure 2); joint effusion and synovitis are also seen. Following the imaging findings, the patient was given a trial of oral colchicine, 0.5 mg, twice daily for three months in conjunction with acetaminophen, 1000 mg. She returned to the clinic for a follow-up after completion of treatment. The patient's progress was evaluated using measures of movement limitation and improvements across these measures. Specific improvements in extension and abduction were noted. Along with an increased improvement in daily activities such as brushing her hair, dressing herself, and successfully raising her arm above shoulder level, reflecting improvements in flexibility, range of motion, and treatment effectiveness. Her pain intensity decreased to a 2 out of 10 on the VAS. Her ESR and CRP levels decreased to 22 mm/hr and 10 mg/L, respectively. Additionally, she experienced no notable adverse effects from the medication.

## Discussion

The case described here demonstrates improved functional activities and reduced pain in glenohumeral OA after a three-month trial of colchicine (0.5 mg, twice a day) with acetaminophen. Pain reduction was more pronounced with continued use by the second and third month. Improvements in VAS scores, ESR and CRP levels were also observed after treatment completion. Colchicine has been previously studied for OA management because of its ability to target shared pathophysiological mechanisms involving neutrophils, elastase and matrix metalloproteinases (MMP), which are often elevated in OA and inhibited by colchicine [9]. We explored the use of colchicine for managing shoulder pain associated with glenohumeral OA, building upon previous evidence of its impact on knee OA and hand OA.

Samuels et al. reported a decrease in VAS scores and improvement in activities in 60 patients with chondrocalcinosis-associated knee OA after a three-month randomized double-blinded clinical trial of daily colchicine (0.5 mg) [10]. Similarly, Das et al. [11] reported a significant 30% improvement in knee pain with crystal evidence, when treated with a combination therapy of IACS injections, NSAID, and colchicine (0.5 mg, twice a day). These outcomes emphasize the beneficial and safe use of colchicine for management of knee OA, specifically involving chondrocalcinosis. Meanwhile, in a randomized double-blinded trial involving 62 patients with primary knee OA, the administration of colchicine (0.5 mg, twice a day) for four months showed improvements in pain, stiffness, and functionality [12]. However, in contrast, Singh et al.'s systematic review and meta-analysis on the impact of colchicine for primary knee OA reported no significant improvements in pain or activity in colchicine and placebo groups of nine randomized clinical trials [13]. These diverse findings suggest that the effects of colchicine may be influenced by the amount of inflammation occurring. When chondrocalcinosis is present, it can exacerbate inflammation, potentially making colchicine more effective in these cases. However, in cases of primary knee OA, inflammation occurs through multiple mechanisms leading to variable outcomes with colchicine use. 

Acute phase reactants like ESR and CRP are used to monitor inflammation but these markers may not always be significant in OA. However, in certain cases, like in the case of our patient, mild elevation may be seen. Colchicine can lower and inhibit CRP via inhibition of nuclear factor kappa B (NF-κB) signaling and NLRP3 inflammasome activation, directly suppressing pro-inflammatory cytokines like IL-6, which inhibit the release of acute phase reactants [14]. Hence why CRP levels may decrease after colchicine therapy, as seen in our patient. Studies have shown that inflammatory markers may be more prominent in erosive type OA. Ertuk et al. conducted a retrospective cohort study to determine whether colchicine therapy, with or without paracetamol, improves pain relief and inflammatory markers in erosive OA of the hand [15]. The study revealed significantly improved ESR and CRP levels, along with VAS scores among participants receiving combination therapy of colchicine and paracetamol, rather than paracetamol alone. These outcomes suggest that colchicine may be most effective in aggressive forms of OA, such as the erosive type, since its pathophysiology involves more pronounced inflammation [15]. In contrast to this, Dossing et al. led a similar trial and had no significant impact on improving the functional activity or pain outcomes in patients with OA of the hand [16]. Though, it’s important to note that erosion was not evident in the studied group of patients. Additionally, the same combination therapy of colchicine and acetaminophen was used in another randomized double-blinded study for primary knee OA, where the studied group of 31 post-menopausal women reported significant improvements in VAS scores compared to acetaminophen use alone [17].

Our patient's imaging findings reveal a pattern of degenerative changes consistent with erosive OA in the glenohumeral joint. This may explain the efficacy of colchicine in our patient's case, since erosive OA involves more pronounced inflammation. This may also explain the slightly elevated inflammatory markers seen in our patient. Furthermore, the previous studies reveal that colchicine may reveal enhanced efficacy when used in a combination therapy approach. In our clinical experience, the combination therapy of colchicine and acetaminophen revealed significant improvements in mobility, VAS scores, ESR and CRP levels for glenohumeral OA. Since there are no reported studies evaluating efficacy of colchicine for glenohumeral OA, our case may encourage potential use of colchicine as an alternative treatment for shoulder pain. 

## Conclusions

This case highlights the incorporation of colchicine for pain management in OA, when more common treatments fail or have contraindications. The administration of colchicine in combination with acetaminophen revealed remarkable improvements in shoulder pain, movement, and inflammatory markers for glenohumeral OA without any adverse effects. While previous research on colchicine use for pain management in OA has shown diverse outcomes, our case helps add to the growing evidence supporting the safety and efficacy of colchicine for OA. To our knowledge, there have been no reported studies for colchicine use specifically in glenohumeral OA, highlighting a gap in the literature. We recommend performing clinical trials and observational studies to explore the potential role of colchicine in glenohumeral OA, and possibly include this as an alternative treatment for pain management. 
